# Colonization and Age Structure of *Dalbulus maidis* Population in Maize Crops During the Vegetative Stage in the Brazilian Cerrado

**DOI:** 10.1007/s13744-026-01388-5

**Published:** 2026-04-21

**Authors:** Tiago Barbosa de Carvalho, Mayara Cristina Lopes, Ricardo de Castro Dias, Givanildo Zildo da Silva, Ricardo Scheffer de Andrade Silva, Daiane das Graças do Carmo, Marcelo Coutinho Picanço

**Affiliations:** 1 Programa de Pós-Graduação Em Produção Vegetal, Univ de Rio Verde, Rio Verde, GO Brazil; 2 Depto de Entomologia, Univ Federal de Viçosa, Viçosa, MG Brazil; 3 Dept of Soil Science, Luiz de Queiroz College of Agriculture, Univ of São Paulo, Piracicaba, Brazil

**Keywords:** Corn leafhopper, Population dynamics, Crop phenology, Climatic factors, Life cycle

## Abstract

*Dalbulus maidis* is a key pest and vector of maize pathogens in Latin America. Understanding the field population dynamics of *D. maidis* is essential for improving management strategies and reducing the risk of pathogen transmission in maize. This study evaluated the population density and stage structure of *D.* *maidis* across maize vegetative stages and determined the effects of crop development and climatic variables on the abundance of eggs, nymphs, and adults over three growing seasons. Field trials were conducted over three consecutive second-crop seasons (2022–2024) in six commercial maize fields in Jataí, Goiás, Brazil. Insect densities (eggs, nymphs, and adults) were monitored every three days using direct plant inspections, and meteorological data were recorded. Insect populations were assessed from V2 to VT stages, and the effects of plant age, phenological stage, and climatic variables were analyzed using multiple regression models. Results showed that *D. maidis* egg and adult densities were higher than nymph densities across all seasons. A consistent pattern was observed wherein early maize stages (V2–V4) favoured oviposition and nymphal development, while adults predominated in later stages (V5–VT). There was a positive and significant correlation between adult density and the age and phenological stage of the plants, while eggs and nymphs did not show a significant correlation with age, phenological stage, or climatic variables. These findings have important implications for Integrated Pest Management (IPM), highlighting a critical window early in crop development when monitoring and control actions are likely to be most effective for preventing population buildup and subsequent pathogen transmission.

## Introduction

The expansion of maize (*Zea mays* L.) cultivation and the reduction in its seasonal planting have altered the dynamics of pest and disease incidence. In this context, the cornleafhopper, *Dalbulus maidis* (DeLong & Wolcott) (Hemiptera: Cicadellidae), has gained prominence, particularly in the Neotropical region (Oliveira and Frizzas 2022). Currently, *D. maidis* is considered the most significant maize pest in Latin America, with yield losses reaching up to 70% (Waquil et al. 1999; Virla et al. 2021; Oliveira and Frizzas 2022; Carmo et al. 2025). Adult *D. maidis* are about 4-mm long, straw-coloured, and have two black spots on their head. The nymphs are yellowish and go through five nymphal stages. The eggs are laid inside the leaf tissue (Waquil et al. 1999). It is a monophagous species (Waquil et al. 1999; Oliveira and Frizzas 2022).

High population densities of *D. maidis* can lead to the wilting and death of young maize plants, primarily due to phloem sap extraction (Waquil et al. 1999; Oliveira and Frizzas 2022). Additional damage may result from the toxic effects of their saliva and the excretion of honeydew, which fosters fungal growth on the foliage (Bushing and Burton 1974; Oliveira and Frizzas 2022; Maluta et al. 2023). However, the most severe damage caused by this pest stems from its role as a vector of multiple maize pathogens. *Dalbulus maidis* transmits *Spiroplasma kunkelii*, the causal agent of maize stunt (CSS), and a phytoplasma associated with maize bushy stunt (MBSP). It is also a vector of maize rayado fino virus (MRFV). These diseases can lead to substantial yield losses in maize production (Virla et al. 2021; Oliveira and Frizzas 2022; Oliveira et al. 2026). Therefore, understanding the population dynamics of *D. maidis* is important given its threat to maize crops and its role as a vector for pathogens.

Pest insect populations fluctuate throughout the year (Farias et al. 2022; Carmo et al. 2025). The population dynamics of this insect are influenced by climatic conditions, the plant’s stage of development, and the control methods applied. Furthermore, it can be influenced by its dispersal capacity and landscape context (Farias et al. 2022; Foresti et al. 2022; Oliveira and Frizzas 2022). Temperature, relative humidity, and rainfall are the primary climatic factors influencing insect pest populations (Silva et al. 2017; Santana et al., 2019; Lopes et al. 2020). During plant development, morphological, physiological, and chemical changes occur that alter the quality of food resources and available habitats, influencing insect oviposition, survival, development, and dispersal (Waltz and Whitham 1997; Spiegel and Price 1996; Farias et al. 2022). Assessing the extent of these influences is crucial for understanding species population dynamics, forecasting pest outbreaks, and designing Integrated Pest Management (IPM) programs. Such programs, incorporating effective sampling and control strategies, play a key role in minimizing economic losses (Naranjo and Ellsworth 2005; Lopes et al. 2020; Farias et al. 2022).

Understanding the seasonal population dynamics of *D. maidis* under field conditions is essential for the development of effective IPM. Information on stage-specific abundance and temporal variation across maize phenological stages and growing seasons is limited. Therefore, this study evaluated the population density and stage structure of *D. maidis* across maize vegetative stages and determined the effects of crop development and climatic variables on the abundance of eggs, nymphs, and adults over three growing seasons. We hypothesized that (i) early vegetative stages would harbour higher proportions of eggs and nymphs due to favourable conditions for oviposition and establishment, (ii) adult abundance would increase as the crop develops, and (iii) temperature would positively affect population density, whereas advanced phenological stages would reduce stage-specific abundance due to declining host suitability.

## Materials and methods

### Study site

**Fig. 1:**
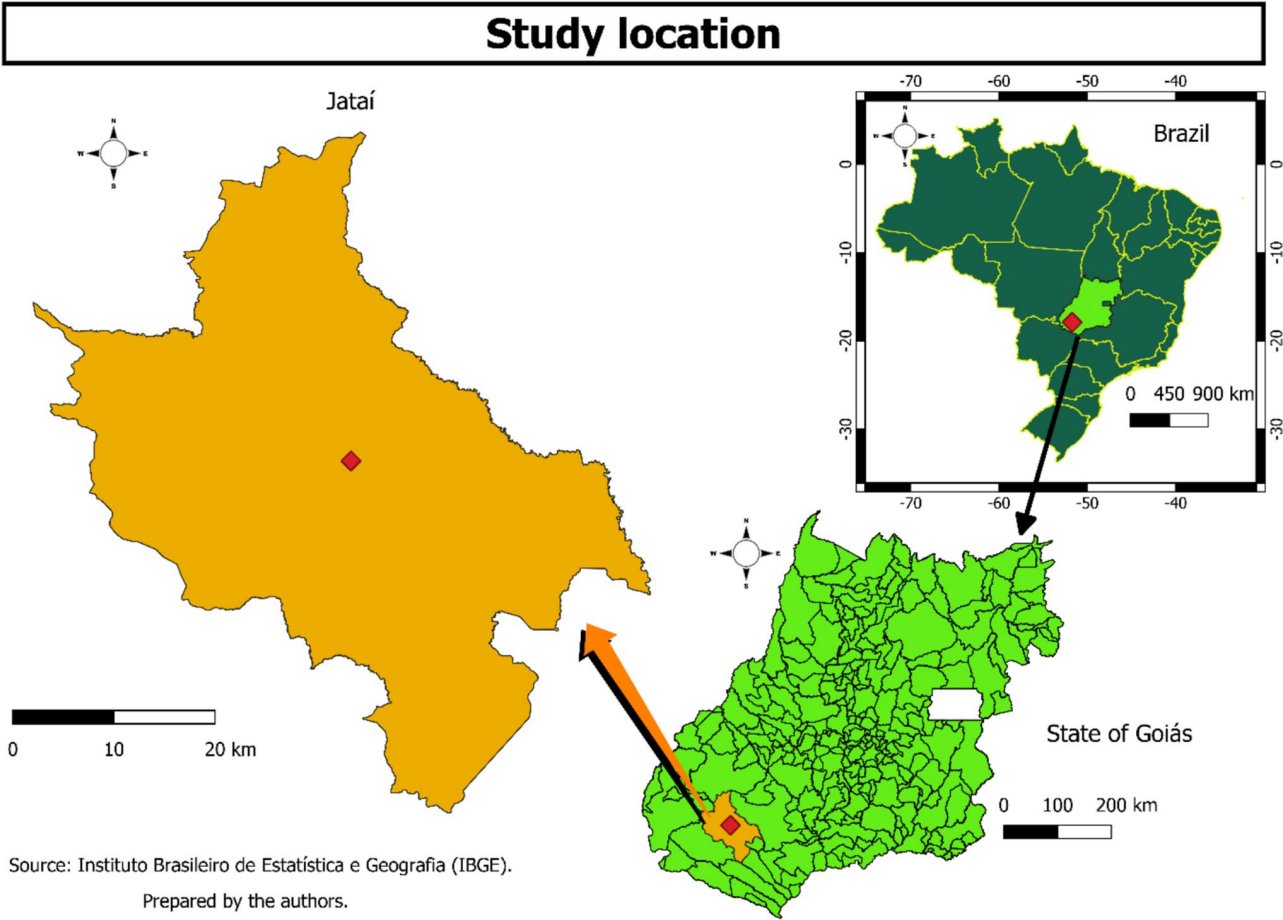
Geographic location of the study area in Jataí, Goiás, Brazil, where the field trials were conducted. The map highlights the municipality within the state and its position in the national context. Data sources: Google Satellite Database and Instituto Brasileiro de Geografia e Estatística (IBGE). Map prepared by the authors

The study was conducted in six commercial maize fields, each with an area of four hectares, located in Jataí, Goiás, Brazil (17°52′53″S, 51°42′52″W; 720 m altitude; tropical climate) (Fig. 1). Field trials were carried out over three consecutive second-crop seasons, from February to April in 2022, 2023, and 2024. Sowing dates were 18 February 2022, 22 February 2023, and 26 February 2024.

The maize cultivar used was BREVANT 2702VYHR, planted at a density of 2.72 plants per meter with 45 cm row spacing under rainfed conditions, totaling 54,400 plants per hectare. Standard agronomic practices were adopted, including fertilizer application based on soil analysis, and the use of pesticides and herbicides for the management of pests, diseases, and weeds. The study was conducted in commercial fields managed by farmers, where insecticides were applied according to local practices. These conditions represent the typical production scenario to which *D. maidis* populations are exposed in maize fields.

Maize plants in the vegetative stage were evaluated. Vegetative stages are designated V*n*, where “*n*” is the number of fully developed leaves produced by the plants. Thus, V1 is determined when the first leaf is fully developed. The last vegetative stage of maize is called VT, when tasselling occurs, allowing the start of reproduction. The maize hybrid BREVANT 2702VYHR typically reaches approximately 18–20 fully expanded leaves.

### Characteristics evaluated

**Fig. 2:**
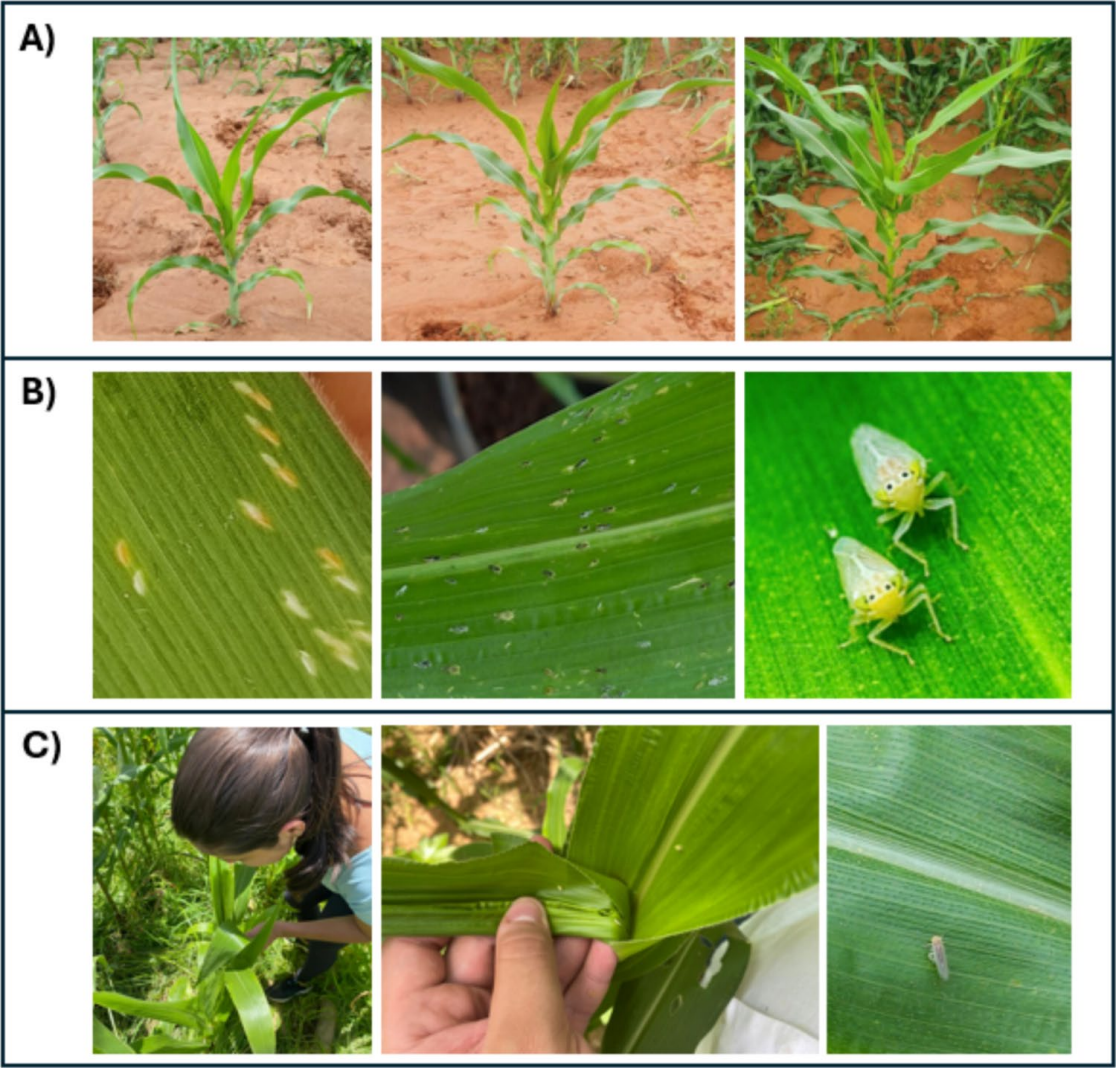
**A** Vegetative growth stages of maize plants monitored during the study, **B** developmental stages of *Dalbulus maidis* on maize leaves, and **C** field sampling of *Dalbulus maidis* populations during plant inspection

The densities of *D. maidis* eggs, nymphs, and adults were evaluated through random sampling of 320 plants per field. Monitoring was conducted every three days throughout the vegetative growth stage (V2 to VT). Insect density was determined through direct visual inspection of the plants. A 60 × magnifying lens was used to assess the presence of eggs and nymphs (Fig. 2). According to Pinto et al. (2023), direct counting of individuals on the plant is a practical, fast, and reliable technique for assessing *D. maidis* populations in maize plantations. To minimize disturbance and potential escape responses, plants were inspected carefully and systematically, and counts were made immediately upon approaching each plant. Meteorological data, including mean air temperature (°C), daily rainfall (mm), and mean relative humidity (%), were obtained from the National Institute of Meteorology (INMET 2024) and cross-validated with data from a weather station installed at the experimental site.

Plant age (expressed as days after emergence) and phenological stage (Vn-VT) were both included in the analyses because they represent complementary aspects of crop development. While plant age captures the temporal progression of the crop under varying environmental conditions, phenological stage reflects structural and morphological changes that may directly influence *D. maidis* colonization, feeding, and reproduction. Considering that phenological development may vary among seasons due to climatic conditions, both variables were analyzed to better disentangle temporal and biological effects on insect population dynamics.

### Statistical analyses

Data analysis was performed using the R software (R Core Team 2025). Data on the densities of eggs, nymphs, and adults of *D. maidis* were subjected to Lilliefors and Cochran tests to assess whether residuals followed and homogeneity of variance, respectively (Cochran 1951; Lilliefors 1967). Since the residuals of the data for these variables did not present a normal probability distribution and homogeneity of variance, the relationships between the densities of eggs, nymphs, and adults of *D. maidis* with the average air temperature, total rainfall, average relative humidity, age, and phenological stage of the maize plants were subjected to Spearman’s correlation analysis. Spearman’s correlation was used because data did not meet normality and homoscedasticity assumptions (Goss-Sampson 2025).

## Results

**Fig. 3:**
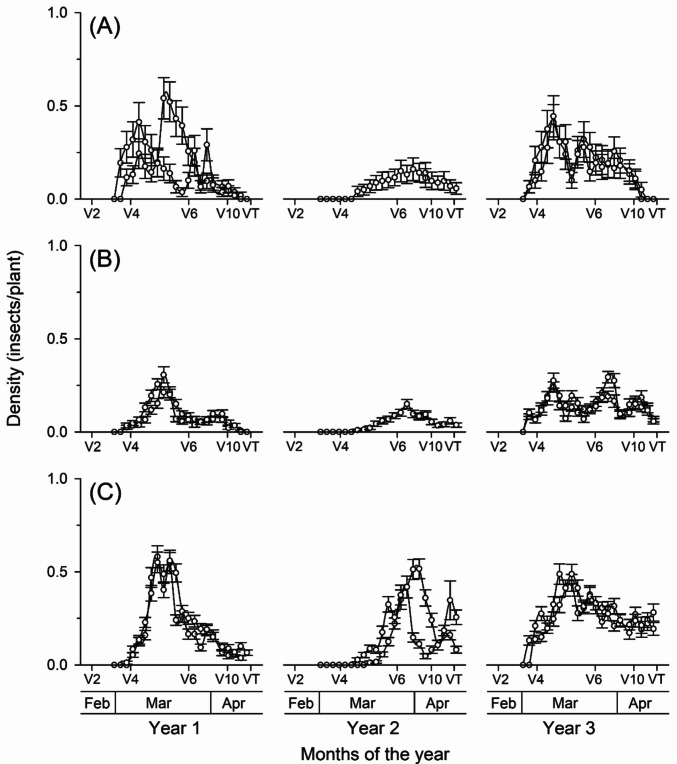
Densities (mean ± standard error) of **A** eggs, **B** nymphs, and **C** adults of *Dalbulus maidis* across maize vegetative stages (V2 to VT) in six commercial fields over three consecutive years

Insect density varied between years and developmental stages (Fig. 3). In Year 1, egg density increased from mid-March, peaking at phenological stages V4–V5, followed by a decline until V10–VT. In Year 2, values remained low and without pronounced peaks. In Year 3, a new population increase was observed, with peaks between V3 and V4 and a reduction in the final stages of the cycle (Fig. 3A). Nymph density in Year 1 showed a moderate peak near stages V4–V5, followed by a gradual decrease. In Year 2, populations remained low during the evaluated period. In Year 3, nymphs exhibited an increase in density, with multiple peaks of intermediate intensity distributed between V4 and V7 (Fig. 3B). Adult *D. maidis* density in Year 1 peaked between stages V4–V5 and subsequently declined. In Year 2, two distinct peaks were observed, between V5 and V7. In the third year, adult densities were higher and more persistent, with a main peak between V4 and V6 and a gradual reduction until V10–VT (Fig. 3C).

**Fig. 4:**
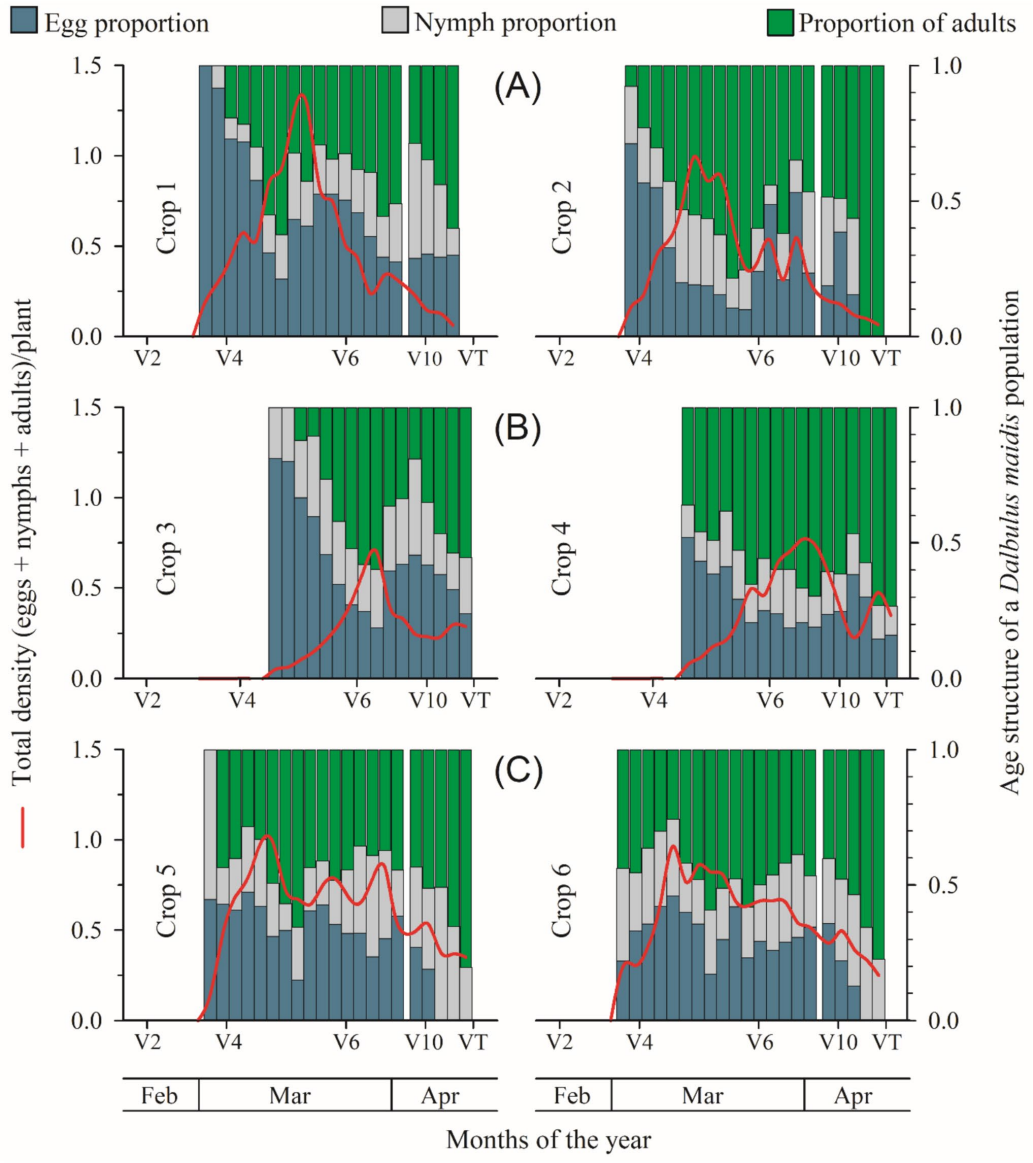
Total density and age structure of *Dalbulus maidis* populations across maize vegetative stages (V2 to VT) in six commercial fields over three years: Year 1 (Crops 1 and 2), Year 2 (Crops 3 and 4), and Year 3 (Crops 5 and 6)

The total density of *D. maidis* and the age structure of the population varied throughout the evaluation period (Fig. 4). The age structure of the population also showed changes over time. In the early stages of the crop, the presence of eggs predominated, followed by a progressive increase in the proportion of nymphs and, later, adults. In the more advanced stages of the cycle, a predominance of adults was observed in most fields. There was an initial increase in population density from the early vegetative stages (V3–V4), with peaks occurring predominantly between V4 and V7, followed by a decline in the later stages (V10–VT). This pattern was consistent in most of the evaluated areas (Fig. 4).

**Fig. 5:**
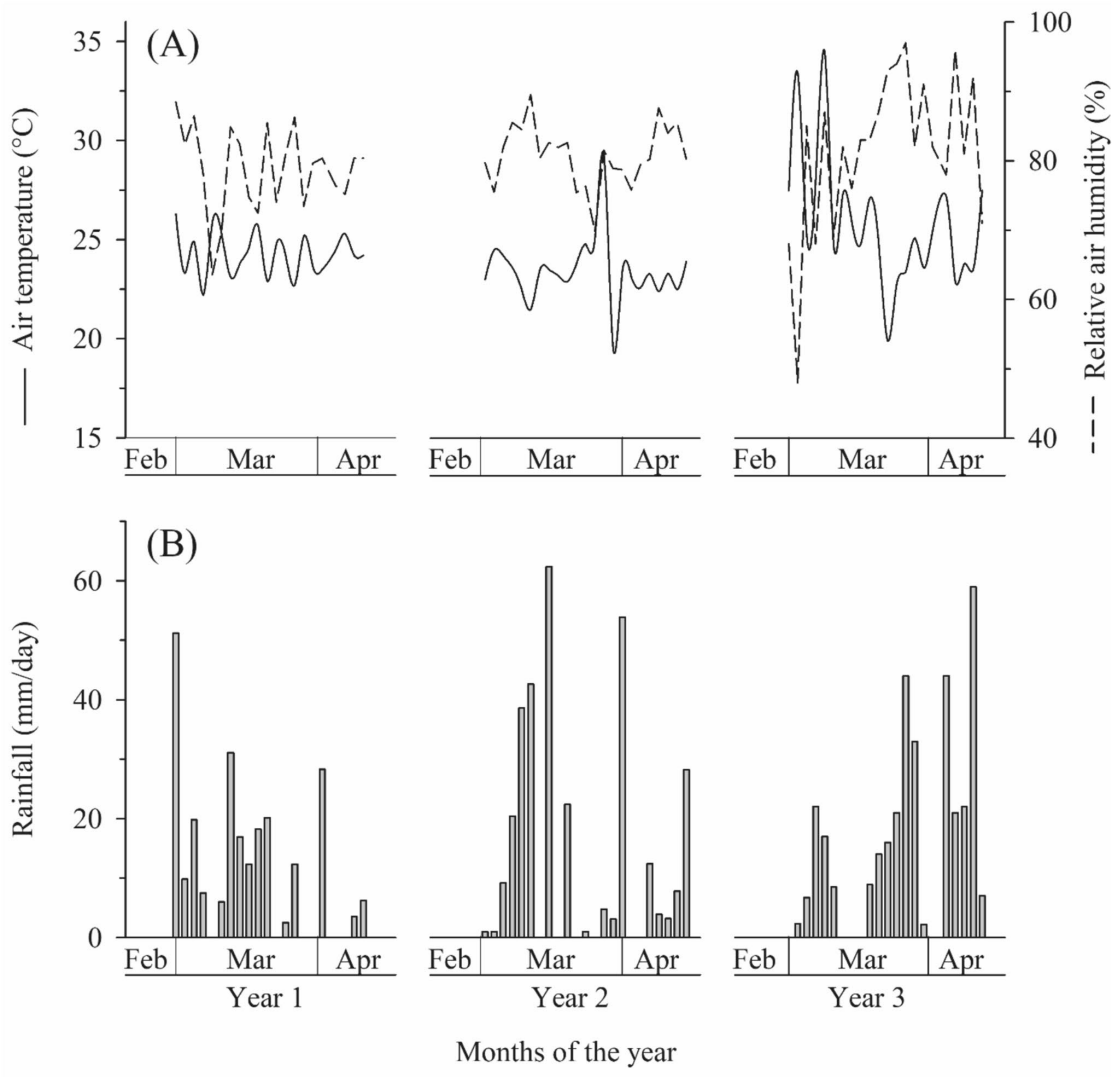
Variation of daily data of **A** average air temperature and average relative air humidity and **B** total rainfall during the period of occurrence of phenological stages V2 to VT of plants in maize crops cultivated in three years

During the experimental period, the average air temperature was 20.98°C, 23.45°C, and 25.8°C in the first, second, and third years respectively. The average relative humidity was 78.4%, 80.77%, and 81.5% in the first, second, and third years respectively. The rainfall was 233.4 mm with a maximum of 51.2 mm/day, 313.9 mm with a maximum of 62.4 mm/day, and 261.6 mm with a maximum of 59.00 mm/day in the first, second, and third years respectively (Fig. 5).

Adult *D. maidis* densities showed significant (*p* < 0.05) and positive correlations with age (*r* = 0.21, *t* = 2.41, df = 132, *p* = 0.0171) and with the phenological stage of the plants (*r* = 0.28, *t* = 3.35, df = 132, *p* = 0.0011). However, correlations between egg and nymph densities of the insect with the age and phenological stage of the plants were not significant (*p* > 0.05). Furthermore, correlations between egg, nymph, and adult densities of the insect with climatic elements were not significant (*p* > 0.05) (Table 1).

**Table 1:** Spearman’s correlations, *t*-test, and significance between egg, nymph, and adult densities of *Dalbulus maidis* and air temperature, rainfall, relative humidity, age, and phenological stage of corn plants

Independent variables	Egg density			Nymph density			Adult density		
	Correlation	*t*	*p*	Correlation	*t*	*p*	Correlation	*t*	*p*
Average air temperature (°C)	0.140	1.63	0.1047	0.042	0.48	0.6307	0.080	0.93	0.3562
Total rainfall (mm/day)	−0.012	−0.14	0.8865	0.084	0.97	0.3362	0.034	0.39	0.7000
Average relative air humidity (%)	0.026	0.30	0.7643	0.163	1.90	0.0601	0.052	0.59	0.5539
Plant age (days)	−0.132	−1.54	0.1261	0.038	0.43	0.6656	0.206	2.41	0.0171
Phenological stage of plants	−0.076	−0.88	0.3823	0.088	1.01	0.3123	0.280	3.35	0.0011

## Discussion

Population fluctuations of key pest species under field conditions are regulated by multiple factors, and understanding these drivers is essential for predicting outbreaks and improving management strategies (Farias et al. 2022; Carmo et al. 2024; Santos et al. 2021). In the case of *D. maidis*, variation in density and age structure across maize phenological stages provides key insights into its establishment and population growth, supporting more efficient monitoring and management strategies.

At the beginning of the evaluations, *D. maidis* densities were low, suggesting that adults may have migrated from nearby fields into the newly established maize area. This behaviour is likely associated with the pest’s preference for ovipositing on young plants (Waquil et al. 1999; Virla et al. 2021; Oliveira and Frizzas 2022; Oliveira et al. 2026). Early vegetative stages were characterized by a predominance of eggs and nymphs, suggesting that young maize plants offer highly favourable conditions for oviposition and the development of immature stages. Young tissues typically offer higher nutritional quality, lower structural defences, and greater accessibility to phloem sap, factors known to improve the performance of phloem-feeding insects (Oliveira et al. 2013; Rossini et al. 2021).

A decline in population density was also observed at the end of the vegetative phase. According to Carmo et al. (2024), *D. maidis* populations tend to decrease as nutrient availability in the host plant declines with crop phenological progression. The observed decline in abundance toward later vegetative stages may also be partially related to early signs of plant senescence. Even before reproductive stages, physiological aging can reduce phloem quality, alter amino acid composition, and increase structural resistance, thereby lowering host suitability for sap-feeding insects (Oliveira et al. 2013; Rossini et al. 2021).

The persistence of *D. maidis* likely depends on dispersal to alternative habitats, survival on volunteer maize plants, or movement among asynchronously planted fields (Oliveira et al. 2013, 2026; Rossini et al. 2021). Such dynamics at the landscape scale may play a critical role in shaping early-season infestations. These findings have important implications for the epidemiology of maize diseases, as adult insects are the primary vectors of phytopathogens associated with corn stunt complexes. Early colonization by infective adults can initiate disease, making the timing of adult arrival a critical determinant of epidemic development (Oliveira et al. 2013, 2026; Rossini et al. 2021; Oliveira and Frizzas 2022).

The positive and significant correlations between adult density and plant age/phenological stage indicate that population increase throughout the cycle results from a combination of continuous immigration of adults from neighboring areas and population growth (Waquil et al. 1999; Oliveira et al. 2013, 2026; Rossini et al. 2021; Oliveira and Frizzas 2022). The arrival of new individuals, particularly in landscapes with staggered plantings, associated with reproduction within the crop itself, promotes population increase. The overlapping of generations, favoured by the maintenance of the host’s physiological quality in vegetative stages, allows eggs, nymphs, and adults to coexist temporally, resulting in a progressive accumulation of individuals throughout the crop's development (Oliveira et al. 2013; Rossini et al. 2021).

The predominance of eggs and nymphs during early vegetative stages indicates that this period is critical for population establishment. Therefore, monitoring and control actions implemented at these stages can effectively reduce population growth and limit disease spread later in the season.

## Conclusions

The results indicate that early crop stages represent a key period for the establishment of *D. maidis* populations, reinforcing the importance of timely monitoring and management interventions to limit population growth and pathogen transmission. In addition, the observed interannual variability highlights the need for continuous surveillance throughout the maize growth cycle. These findings support the development of more dynamic and season-specific integrated pest management strategies.

## Data Availability

The data generated during and/or analyzed during the current study are available from the corresponding author on reasonable request.
